# World vitiligo day: a model for grassroots medical activism and pharmaceutical innovation

**DOI:** 10.3389/fmed.2025.1571422

**Published:** 2025-04-15

**Authors:** Y. Valle, C. M. Arenas Soto, A. A. Kassymkhanova, J. Ocampo-Candiani, S. P. Gondokaryono, I. Binić, N. V. Thuong, P. R. Cunha, J. Hercogová, X. H. Gao, D. Parsad, H. W. Lim, T. Lotti, J. Sigova

**Affiliations:** ^1^Vitiligo Research Foundation, New York, NY, United States; ^2^Department of Dermatology, University of Studies Guglielmo Marconi, Rome, Italy; ^3^Department of Dermatology, Federico Lleras Acosta Dermatological Center, Universidad Militar Nueva Granada, Bogota, Colombia; ^4^Vitiligo Center, Almaty, Kazakhstan; ^5^Hospital Universitario Dr. José e. Gonzalez, Universidad Autónoma de Nuevo León, Monterrey, Mexico; ^6^Department of Dermatology and Venereology, Faculty of Medicine, Universitas Padjadjaran, Bandung, Indonesia; ^7^Medical Faculty, University of Niš, Niš, Serbia; ^8^Vietnam National Hospital of Dermatology and Venereology, Hanoi, Vietnam; ^9^Department of Dermatology, Jundiaí Medical School, São Paulo, Brazil; ^10^Institute of Clinical and Experimental Medicine, Prague, Czechia; ^11^Department of Dermatology, The First Hospital of China Medical University, Shenyang, China; ^12^Department of Dermatology, Venereology and Leprology, Postgraduate Institute of Medical Education and Research, Chandigarh, India; ^13^Department of Dermatology, Henry Ford Hospital, Detroit, MI, United States; ^14^Department of Neonatology, Faculty of Continued Medical Education of Pirogov Russian National Research Medical University, Moscow, Russia

**Keywords:** vitiligo, awareness, patient support, advocacy, drug development

## 1 Introduction

World Vitiligo Day (WVD) is a transformative model of medical advocacy, demonstrating how patient-led initiatives can shift public perception, empower patients, influence legal frameworks, and attract pharmaceutical investment.

The WVD Presidential Committee, comprising this paper's co-authors, asserts that WVD has evolved from a grassroots movement into a global force, reshaping public discourse on vitiligo and driving unprecedented pharmaceutical interest. By analyzing WVD's structure, international reach, and broader impact, we propose it as a replicable framework for future medical advocacy. This model can be adapted for other neglected diseases, ensuring greater visibility, research funding, and policy engagement.

## 2 Discussion

### 2.1 Origins and global expansion of WVD

WVD emerged from independent advocacy initiatives spanning multiple regions (1). In India, Dr. Savita Malhotra established National Vitiligo Day on May 19, observed from 2009 to 2015, until Prof. Davinder Parsad successfully unified three national dermatological societies to endorse WVD. Independently, in the United States, Valarie Molyneaux of VITFriends and other patient advocates promoted a national vitiligo awareness day without specifying a particular date.

A major precursor to WVD took place on June 25, 2011, when Ogo Maduewesi of VITSAF organized the “Purple Fun Day” event in Nigeria. Held on the anniversary of Michael Jackson's passing—recognizing his lifelong struggle with vitiligo (2)—the event sought to raise awareness and combat the stigma surrounding the condition in Africa.

Recognizing the opportunity for global coordination, the U.S.-based non-profit Vitiligo Research Foundation (VRF) formally established June 25 as World Vitiligo Day. The first internationally coordinated WVD was convened in 2012 during the press conference at the University of Guglielmo Marconi in Rome, under the leadership of Prof. Torello Lotti and Yan Valle.

A defining feature of WVD's success is its rolling headquarters model, in which the host country rotates annually to maximize global engagement. Host nations between 2012 and 2025 include: Italy, India, China, Czech Republic, Brazil, USA, Vietnam, Serbia, Indonesia, Mexico, Kazakhstan, Colombia, and Canada.

At the national level, WVD adapts to regional customs: for example, in the USA and European Union (EU), events rotate among different states or cities annually, while in China and India, activities are consistently held in familiar locations. This adaptive framework has sustained global growth while reinforcing regional advocacy initiatives.

### 2.2 Milestones, policy influence, and global impact

Initially, the WVD Committee aimed to collect 500,000 petition signatures to secure UN recognition. This effort was not merely about numbers; it was a strategic initiative designed to encourage governments to leverage UN and WHO resources to boost research efforts. The WVD campaign drew inspiration from World Psoriasis Day, which later successfully attained WHO recognition in 2014 after a decade of sustained advocacy (3).

These efforts yielded results when WVD reached the target and received a formal nod from the United Nations' Economic and Social Council (UN ECOSOC) committee in mid-2016, being marked on the UN Calendar of Disability Events—signaling an opportunity for future discussions on official recognition (4). However, in 2019, a revision of the calendar to feature only “Major Events” led to a change in WVD's status. Efforts to reinstate WVD on the UN calendar were hindered by the COVID-19 pandemic and its widespread disruptions.

Recent findings indicate that specific “health days” can provide greater impact than “health months.” A study on awareness campaigns showed that World Psoriasis Day (October 29) garnered higher public engagement than Psoriasis Action Month (August) (5). The same thesis has been independently confirmed by analysis of PCOS (polycystic ovary syndrome) awareness campaign outcomes (6). These findings reinforce that WVD's single-day approach may be particularly effective in achieving campaign objectives. Notably, WVD-2022 events in Mexico demonstrated a digital reach exceeding that of comparable dermatological initiatives (7).

### 2.3 A decentralized and networked model of WVD

Unlike traditionally hierarchical disease awareness campaigns led by major health organizations, WVD follows a networked, decentralized structure. At the core, the WVD Presidential Committee provides strategic direction while nurturing an agile and engaged network of activists. It also oversees the annual WVD conference, which brings together up to 300 healthcare professionals for an intensive one- to three-day event in the host country.

Local groups maintain autonomy, allowing them to tailor campaigns to regional cultural contexts while benefiting from global coordination. By leveraging mass media, social media, grassroots advocacy networks, and dermatology societies, WVD has mobilized millions—without reliance on a centralized governing entity.

This bottom-up model diverges from conventional health awareness campaigns, which are often characterized by top-down governance and institutional sponsorship from major non-profits or dermatology associations. While organizations such as the World Health Organization (WHO) and the Health Campaign Effectiveness Coalition advocate for centralized coordination (8), WVD's decentralized model has demonstrated its ultimate cost-effectiveness in fostering culturally sensitive outreach across diverse communities.

WVD events range from medical conferences to community support groups and artistic exhibitions, adapting to local needs and resources. This flexibility has enhanced WVD's ability to drive systemic change—leading to official recognition by numerous U.S. city mayors and state governors, as well as influencing major insurance companies' coverage policies (9, 10).

### 2.4 Pharmaceutical engagement and market expansion

One of WVD's most significant achievements has been its role in catalyzing pharmaceutical investment in vitiligo. When WVD was initiated in 2012, vitiligo remained largely overlooked by the pharmaceutical industry, with the market size for vitiligo therapeutics being negligible. Recognizing this gap, an implicit objective of the campaign was to establish vitiligo as a viable focus for pharmaceutical investment. This involved actively engaging industry representatives at every WVD meeting and ensuring the FDA gained a comprehensive understanding of the vitiligo patient experience—an effort that has demonstrated its efficacy.

The impact of WVD was exemplified in early 2021 when the FDA convened its inaugural Patient-Focused Drug Development Meeting on vitiligo (11). By then, more than forty pharmaceutical and biotechnology companies had entered the field, with one drug approved by both the FDA and EMA (European Medicines Agency) in 2022–2023. Currently, multiple others are progressing through clinical trials (12), driving estimates of the global vitiligo treatment market to $2.76 billion by 2032 (13).

This surge in industry attention and market growth can, at least in part, be attributed to WVD's ability to spotlight vitiligo's psychosocial and economic impact. Through themed events, mass-media publications, and strategic advocacy, WVD has made vitiligo a compelling area for research and investment. WVD illustrates the capacity of patient-driven movements to influence research priorities, regulatory frameworks, and pharmaceutical innovation—transforming vitiligo from a neglected condition into a rapidly expanding treatment market.

### 2.5 Future directions and challenges

Looking ahead, WVD faces both opportunities and challenges. The increasing recognition of vitiligo as a serious medical condition presents concrete opportunities, such as influencing healthcare policies and securing dedicated research funding. However, maintaining the campaign's grassroots ethos amid rapid global growth presents challenges, particularly managing local autonomy vs. global consistency (13).

A standardized yet adaptable framework is being developed for the WVD-2025 campaign, with headquarters set in Toronto, Canada, combining global strategic oversight with sensitivity to local cultural contexts and legislative frameworks. This framework includes clearly outlined “WVD Event Terms and Conditions” and a comprehensive “WVD Events Code of Conduct”.

Historically, each WVD headquarters created its own local website while the WVD committee maintained the main website at 25June.org. This allowed local talent to significantly contribute to campaign's website and content development, respecting vastly diverse cultural contexts across past events held in the Americas, Africa, Asia, and Europe—but also introduced inconsistencies in user experiences and technical compatibility issues. For WVD-2025, a standardized yet customizable website template has been developed to ensure consistency across campaigns, while allowing flexibility for annual themes and unique design elements in the future.

The 2025 theme, “Innovation for Every Skin, Powered by AI,” emphasizes harnessing AI to democratize access to accurate and supportive vitiligo information. Overall, the 2025 campaign efforts focus on:

1. Expanding media outreach into underserved provinces and regions, especially considering Canada's lengthy average wait time (exceeding 8 months) to see a dermatologist.
2. Incorporating an existing AI-powered digital assistant (vitiligo.ai) offering multilingual support, educational resources, and personalized engagement tools, thus strengthening connections within the vitiligo community while preserving personal autonomy.
3. Strengthening collaborations between national patient support group, individual advocates and researchers to align research priorities more closely with patient needs.

Successfully addressing these challenges will enable WVD to further refine its advocacy model, ensuring broader global recognition and sustained impact in the evolving landscape of health awareness.

Patient voices and associated privacy concerns remain paramount, especially as storytelling and social media outreach grow. Each global WVD event aims to establish or strengthen national support groups, and WVD-2025 is no exception, with Vitiligo Voices Canada having representation on the organizational committee and a dedicated session within the main conference.

### 2.6 Pediatric and young adult focus

Growing evidence highlights substantial psychosocial impacts of vitiligo on children and adolescents, including increased vulnerability to bullying, anxiety, and depression. WVD is committed to addressing these issues through targeted educational initiatives in preschools and schools, including interactive workshops and awareness sessions designed to promote understanding and inclusion. Additionally, the WVD organizing committee will distribute age-appropriate books about vitiligo, in both physical and electronic formats, to up to 50 libraries across Canada, aiming to boost awareness, improve children's self-esteem, and reduce stigma around the condition.

## 3 Conclusions

World Vitiligo Day (WVD) redefines traditional health advocacy through its hybrid model of grassroots mobilization and decentralized governance. By empowering independent teams within a unified strategic framework, WVD has achieved transformative outcomes:

1. Shifted global discourse on vitiligo from stigma to medical urgency.
2. Elevated patient needs and disease prioritization in global health agendas.
3. Catalyzed pharmaceutical innovation through targeted industry engagement.
4. Reshaped international legislative and insurance landscapes.

The campaign's success dismantles the perception of awareness days as performative gestures, demonstrating their capacity to drive systemic change. As WVD prepares for its 14th annual observance in 2025, its model provides a replicable blueprint for addressing underrepresented medical conditions. This framework bridges advocacy efforts with clinical research priorities, creating actionable pathways for democratizing medical progress through participatory action.
